# Supplementary material for: Visual feedback and motor memory contributions to sustained motor control deficits in autism spectrum disorder across childhood and into adulthood
Source: J Neurodev Disord. 2025 May 16;17:26. doi: 10.1186/s11689-025-09607-7 (PMC12083110; doi:10.1186/s11689-025-09607-7)
Supplement: Supplementary file 1 — Supplementary Material 1 [file 11689_2025_9607_MOESM1_ESM.docx]

**Supplementary Material**

**Methods**

To better understand force accuracy, variability, and regularity differences between the ASD and NT groups across ages, *post hoc* analyses were conducted replacing age as a continuous variable in our final models with age as categorical variable (i.e., age bins) in our linear mixed effects models (MLMs). Age bins were determined using a median split of age in the full sample. The median age is 13.9 years.

**Results**

*Force Accuracy (with age bins)*

The model summary force accuracy with age as a categorical variable is reported in Table S1. With age as a categorical variable, the group x age bin interaction was not significant. This was a difference from the results of the model that included age as a categorical variable. Across ages and groups, force accuracy was greater in the visually guided condition relative to the memory guided condition (β = -.22, R^2^ = 0.536, t_75.9_ = -15.56, p < 0.0001), consistent with the model that included age as a continuous variable. The autistic individuals showed lower accuracy than NT individuals (β = -.05, R^2^ = 0.045, t_77.0_ = -2.34, p = .022; mean_ASD_ = .80 ± .01, mean_NT_ = .84 ± .02) and force accuracy was greater in the older relative to the younger age bin (β = .05, R^2^ = 0.039, t_77.8_ = 2.16, p = .034; mean_younger_ = .80 ± .02, mean_older_ = .84 ± .01), consistent with the model that included age as a continuous variable.

**Table S1.** Linear mixed effects model summary for force accuracy with age as a categorical variable

|  | **Fixed Effects** | **Estimate (SE)** | **df** | **t** | **Partial R^2^** |
| --- | --- | --- | --- | --- | --- |
| **Accuracy** | *Intercept* | .82 (.01) | 76.8 | **81.45***** |  |
|  | *Level 1* |  |  |  |  |
|  | Condition | -.22 (.01) | 75.9 | **-15.56***** | .536 |
|  | Load Cells | -.03 (.02) | 78.2 | -1.45 | .018 |
|  | *Level 2* |  |  |  |  |
|  | Group | -0.05 (.02) | 77.0 | **-2.34*** | .045 |
|  | Age Bin | .05 (.02) | 77.8 | **2.16***** | .039 |
|  | Sex | .01 (.02) | 76.9 | .62 | .003 |
|  | *Interactions* |  |  |  |  |
|  | Group x Age Bin | .05 (.04) | 77.8 | 1.20 | .012 |
|  | **Random Effects** | **Variance (SD)** |  |  |  |
|  | *Participant (intercept)* | .003 (.056) |  |  |  |
|  | Residual | .007 (.086) |  |  |  |

SD: Standard Deviation; SE: standard error. * p < .05, ** p < .01, *** p < .001

*Force Variability (with age bins)*

Results of the model for force SD with age as a categorical variable are summarized in **Table S2**. There was a significant group x age bin interaction (β = -0.43, R^2^ = .132, t_75.8_ = -3.89, p = 0.0002), consistent with the results of the model that included age as a continuous variable. Follow-up comparisons revealed that the ASD group showed greater force SD than the NT control group only in the young age bin (mean_ASD_ = .27 ± .05, mean_NT_ = -0.02 ± .07). Overall, the ASD group showed higher force SD than the NT control group (β = 2.41, R^2^ = .131, t_77.5_ = 3.89, p = 0.0002). Sex was a significant covariate (β = -0.16, R^2^ = .069, t_74.9_ = -2.71, p = 0.008), such that males showed greater force variability than females (mean_M_ = .23±.04; mean_F_ = .06 ± .04), consistent with the model that included age as a continuous variable. The effect of load cells reached significance in this model (β = .14, R^2^ = .051, t_76.2_ = 2.31, p = 0.024), whereas it was not significant in the model with age as a continuous variable. Follow-up analyses revealed that Honeywell load cells showed higher force SD than ELFF load cells (mean_ELFF_ = .07 ± .05 , mean_Honeywell_ = .21 ± .03).

**Table S2.** Linear mixed effects model summary for force variability (SD) with age as a categorical variable

|  | **Fixed Effects** | **Estimate (SE)** | **df** | **t** | **Partial R^2^** |
| --- | --- | --- | --- | --- | --- |
| **SD (log_10_)** | *Intercept* | .14 (.03) | 75.1 | **4.99***** |  |
|  | *Level 1* |  |  |  |  |
|  | Condition | .04 (.03) | 70.7 | 1.34 | .006 |
|  | Load Cells | .14 (.06) | 76.2 | **2.31*** | .051 |
|  | *Level 2* |  |  |  |  |
|  | Group | .07 (.06) | 75.2 | 1.32 | .017 |
|  | Age Bin | .03 (.06) | 75.2 | .54 | .003 |
|  | Sex | -0.16 (.06) | 74.9 | **-2.71**** | .069 |
|  | *Interactions* |  |  |  |  |
|  | Group x Age Bin | -0.43 (.11) | 75.8 | **-3.89***** | .132 |
|  | **Random Effects** | **Variance (SD)** |  |  |  |
|  | *Participant (intercept)* | .04 (.19) |  |  |  |
|  | Residual | .03 (.18) |  |  |  |

SD: standard deviation; SE: standard error. * p < .05, ** p < .01, *** p < .001

*Force Regularity (with age bins)*

Results of the model for force SampEn with age as a categorical variable are summarized in **Table S3**. There were significant group x task condition (β = .13, R^2^ = .028, t_75.1_ = 2.60, p = 0.011) and group x age bin interactions (β = .18, R^2^ = .047, t_78.6_ = 2.32, p = 0.023), consistent with the model that included age as a continuous variable. Follow-up comparisons revealed that the ASD group showed a lower force SampEn than the NT control group only in the young age bin (mean_ASD_ = -0.84 ± .04, mean_NT_ = -0.67 ± .05), and the ASD group showed lower SampEn than NT controls only in the visually guided condition (mean_ASD_ = -0.63 ± .03, mean_NT_ = -0.49 ± .04). The task condition x age bin interaction was not significant.

**Table S3.** Linear mixed effects model summary for force regularity (SampEn) with age as a categorical variable

|  | **Fixed Effects** | **Estimate (SE)** | **df** | **t** | **Partial R^2^** |
| --- | --- | --- | --- | --- | --- |
| **SampEn (log_10_)** | *Intercept* | -0.70 (.02) | 77.8 | **-35.88***** |  |
|  | *Level 1* |  |  |  |  |
|  | Condition | -0.28 (.03) | 75.3 | **-11.11***** | .342 |
|  | Load Cells | .01 (.04) | 78.6 | .25 | .001 |
|  | *Level 2* |  |  |  |  |
|  | Group | -0.07 (.04) | 77.9 | -1.93 | .033 |
|  | Age Bin | .12 (.04) | 77.9 | **2.75**** | .065 |
|  | Sex | -0.008 (.04) | 77.5 | .245 | < .001 |
|  | *Interactions* |  |  |  |  |
|  | Group x Condition | .13 (.05) | 75.1 | **2.60*** | .028 |
|  | Group x Age Bin | .18 (.08) | 78.6 | **2.32*** | .047 |
|  | Condition x Age Bin | -0.06 (.05) | 76.1 | -1.30 | .007 |
|  | **Random Effects** | **Variance (SD)** |  |  |  |
|  | *Participant (intercept)* | .01 (.11) |  |  |  |
|  | Residual | .02 (.15) |  |  |  |

SampEn: Sample Entropy; SD: Standard Deviation; SE: standard error. * p < .05, ** p < .01, *** p < .001

**Supplementary Figures**

**
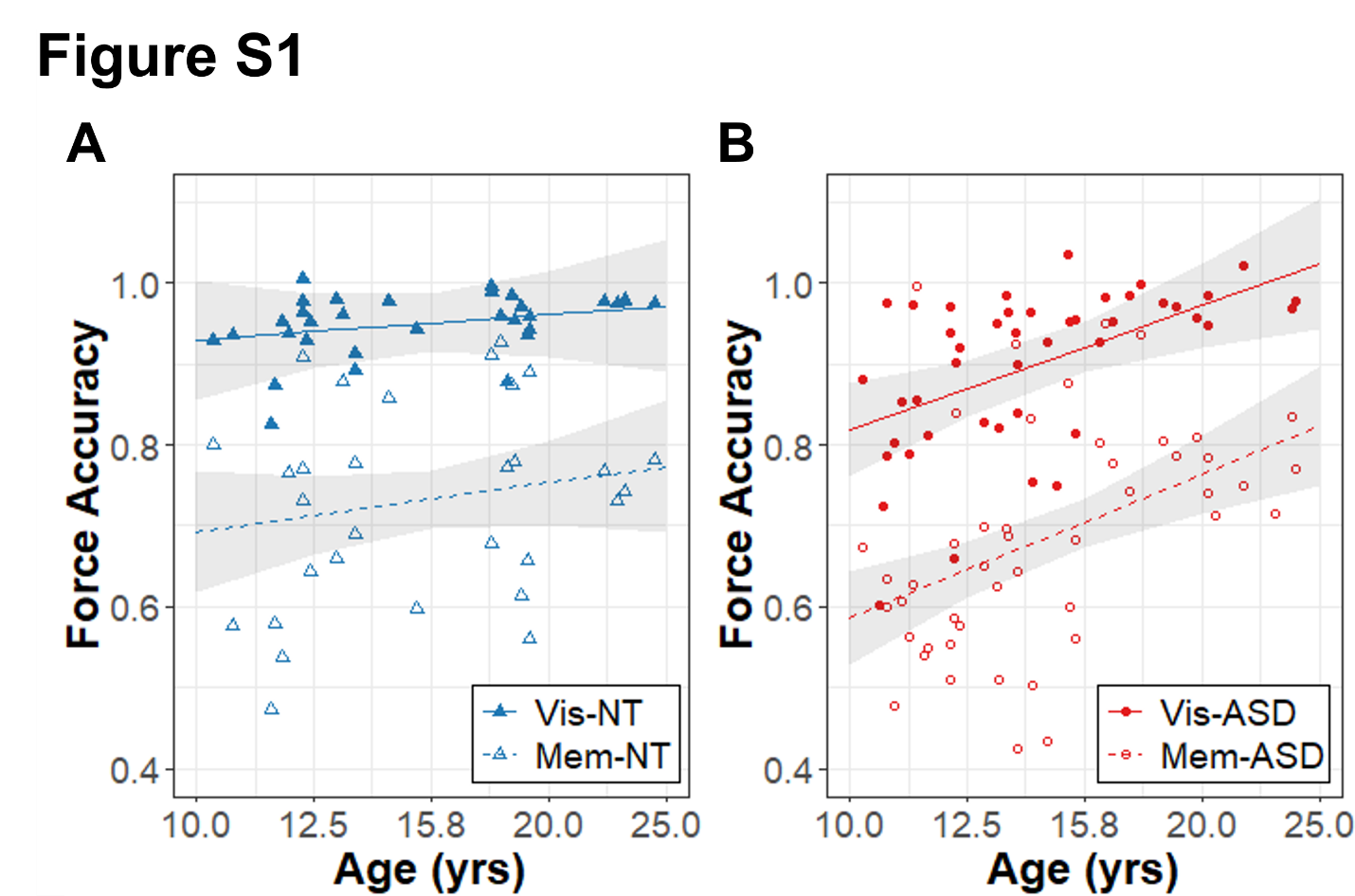
**

**Figure S1. Force accuracy as a function of age, group, and condition.** Age (years; log_10_ scale) associations with force accuracy (proportion of mean force to target force) for **A)** the autism group (ASD; red circles) during visually guided (Vis; solid points) and memory guided (Mem; empty points) precision gripping and **B)** the neurotypical group (NT; blue triangles) during visually guided (Vis; solid points) and memory guided (Mem; empty points) precision gripping. The regression lines represent the group x age x condition trends, though the 3-way interaction was not significant. Error bands represent the 95% confidence intervals from the MLM models after accounting for random intercepts of participant.

**
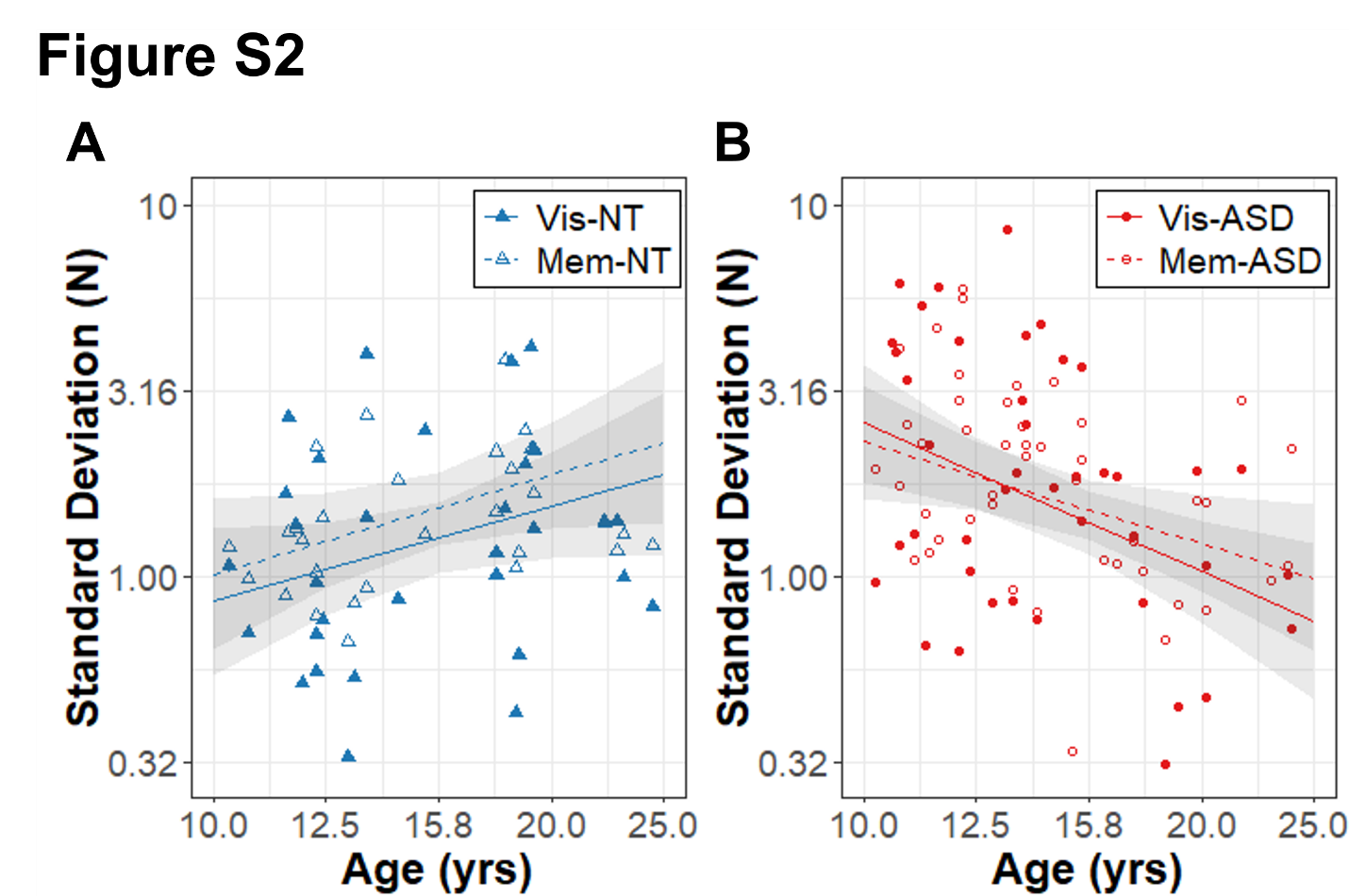
**

**Figure S2. Force Variability in Newtons (N) as a function of age, group, and condition.** Age (years; log_10_ scale) associations with force standard deviation in Newtons (N; log_10_ scale) for **A)** the autism group (ASD; red circles) during visually guided (Vis; solid points) and memory guided (Mem; empty points) precision gripping and **B)** the neurotypical group (NT; blue triangles) during visually guided (Vis; solid points) and memory guided (Mem; empty points) precision gripping. The regression lines represent the group x age x condition trends, though the 3-way interaction was not significant. Error bands represent the 95% confidence intervals from the MLM models after accounting for random intercepts of participant.


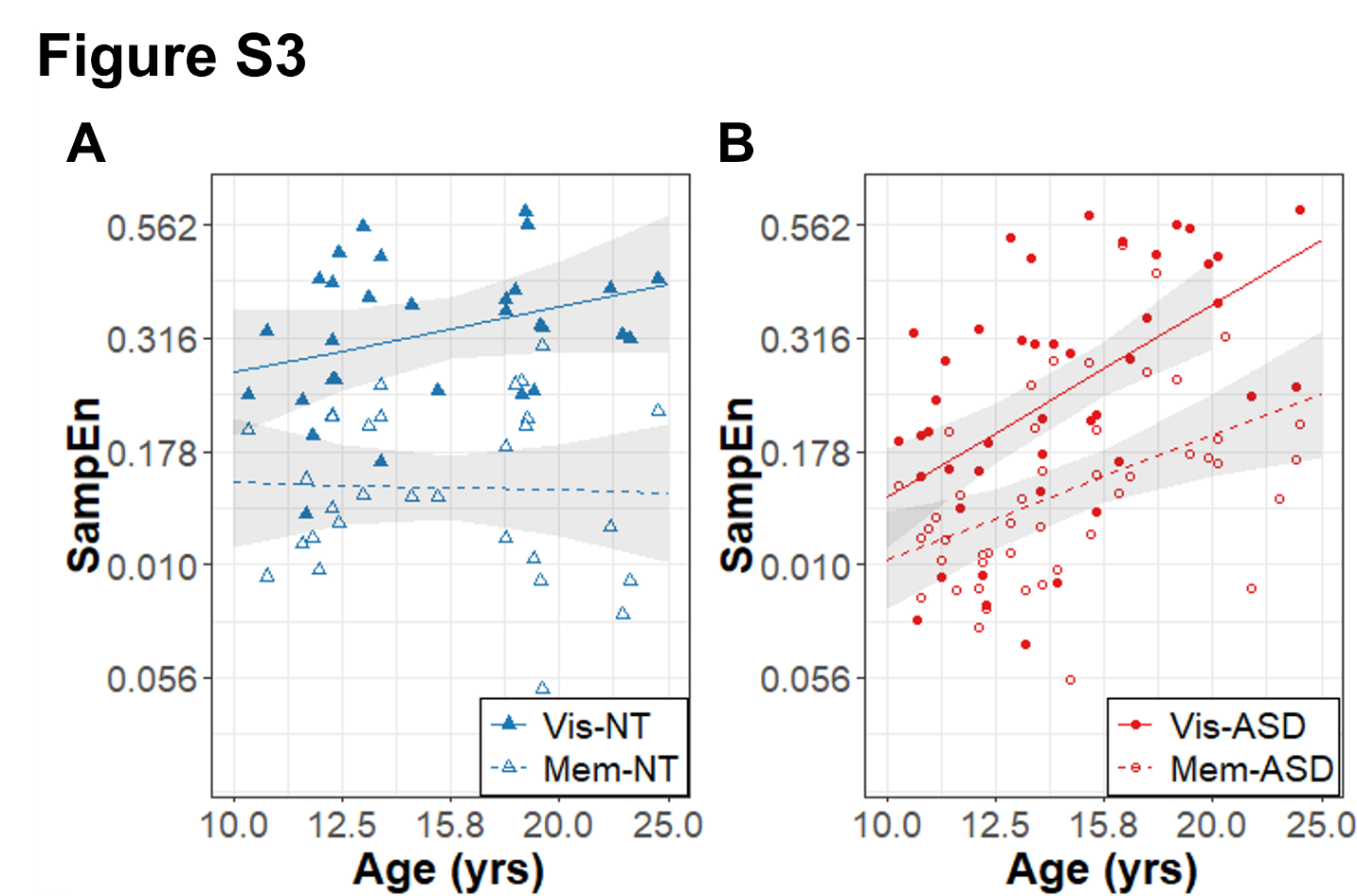


**Figure S3. Force Regularity as a function of age, group, and condition.** Age (years; log_10_ scale) associations with force SampEn (unitless; log_10_ scale) for **A)** the autism group (ASD; red circles) during visually guided (Vis; solid points) and memory guided (Mem; empty points) precision gripping and **B)** neurotypical group (NT; blue triangles) during visually guided (Vis; solid points) and memory guided (Mem; empty points) precision gripping. Higher SampEn corresponds to lower regularity. The regression lines represent the group x age x condition trends, though the 3-way interaction was not significant. Error bands represent the 95% confidence intervals from the MLM models after accounting for random intercepts of participant.
